# TLR2 and caspase-1 signaling are critical for bacterial containment but not clearance during craniotomy-associated biofilm infection

**DOI:** 10.1186/s12974-020-01793-6

**Published:** 2020-04-14

**Authors:** Amy L. Aldrich, Cortney E. Heim, Wen Shi, Rachel W. Fallet, Bin Duan, Tammy Kielian

**Affiliations:** 1 Department of Pathology and Microbiology, University of Nebraska Medical Center, 985900 Nebraska Medical Center, Omaha, NE 68198 USA; 2 Present Address: Moffitt Cancer Center, Tampa, FL 33612 USA; 3 Mary and Dick Holland Regenerative Medicine Program, Division of Cardiology, Department of Internal Medicine, University of Nebraska Medical Center, Omaha, NE 68198 USA

**Keywords:** Craniotomy, *S. aureus*, Biofilm, Toll-like receptor, Caspase-1, IL-1β, Microglia, Macrophage, Myeloid-derived suppressor cell

## Abstract

**Background:**

A craniotomy is required to access the brain for tumor resection or epilepsy treatment, and despite precautionary measures, infectious complications occur at a frequency of 1–3%. Approximately half of craniotomy infections are caused by *Staphylococcus aureus* (*S. aureus*) that forms a biofilm on the bone flap, which is recalcitrant to antibiotics. Our prior work in a mouse model of *S. aureus* craniotomy infection revealed a critical role for myeloid differentiation factor 88 (MyD88) in bacterial containment and pro-inflammatory mediator production. Since numerous receptors utilize MyD88 as a signaling adaptor, the current study examined the importance of Toll-like receptor 2 (TLR2) and TLR9 based on their ability sense *S. aureus* ligands, namely lipoproteins and CpG DNA motifs, respectively. We also examined the role of caspase-1 based on its known association with TLR signaling to promote IL-1β release.

**Methods:**

A mouse model of craniotomy-associated biofilm infection was used to investigate the role of TLR2, TLR9, and caspase-1 in disease progression. Wild type (WT), TLR2 knockout (KO), TLR9 KO, and caspase-1 KO mice were examined at various intervals post-infection to quantify bacterial burden, leukocyte recruitment, and inflammatory mediator production in the galea, brain, and bone flap. In addition, the role of TLR2-dependent signaling during microglial/macrophage crosstalk with myeloid-derived suppressor cells (MDSCs) was examined.

**Results:**

TLR2, but not TLR9, was important for preventing *S. aureus* outgrowth during craniotomy infection, as revealed by the elevated bacterial burden in the brain, galea, and bone flap of TLR2 KO mice concomitant with global reductions in pro-inflammatory mediator production compared to WT animals. Co-culture of MDSCs with microglia or macrophages, to model interactions in the brain vs. galea, respectively, also revealed a critical role for TLR2 in triggering pro-inflammatory mediator production. Similar to TLR2, caspase-1 KO animals also displayed increased *S. aureus* titers coincident with reduced pro-inflammatory mediator release, suggestive of pathway cooperativity. Treatment of caspase-1 KO mice with IL-1β microparticles significantly reduced *S. aureus* burden in the brain and galea compared to empty microparticles, confirming the critical role of IL-1β in limiting *S. aureus* outgrowth during craniotomy infection.

**Conclusions:**

These results demonstrate the existence of an initial anti-bacterial response that depends on both TLR2 and caspase-1 in controlling *S. aureus* growth; however, neither pathway is effective at clearing infection in the WT setting, since craniotomy infection persists when both molecules are present.

## Introduction

A craniotomy is required to access the brain during neurosurgical procedures, such as tumor resection or epilepsy treatment, where the piece of resected skull, or bone flap, is replaced intraoperatively. Although numerous approaches are employed to prevent infectious complications following craniotomy, infections occur in 1–3% of patients [1]. Approximately one half of craniotomy infections are attributed to the gram-positive pathogen *Staphylococcus aureus* (*S. aureus*), which forms a biofilm on the bone flap surface [2, 3]. Biofilms are bacterial communities that are defined by their recalcitrance to immune-mediated clearance and antibiotics, due in part, to the presence of a sub-population of metabolically dormant persister cells [4]. Therefore, craniotomy infections usually require a second procedure for treatment, with one of two surgical approaches typically employed. One is to salvage the native bone flap intraoperatively through aggressive debridement and immersion in an antiseptic followed by a prolonged course of i.v. and oral antibiotics to eliminate residual bacteria [5–7]. Although several reports describing this approach exist, they involve small patient cohorts and the decision of whether to salvage the bone flap remains with the neurosurgeon. A second treatment strategy is to discard the infected bone flap, and after a variable treatment period of i.v. and oral antibiotics spanning from weeks to months, a cranioplasty is performed to seal the cranium with either an alloplastic implant or bone graft [8]. In both scenarios, infections can recur, which underscores the need to understand how bacterial biofilms subvert immune-mediated clearance to establish persistent infection.

Our laboratory has developed a mouse model of *S. aureus* craniotomy infection that shares attributes of human disease, including similarities in biofilm structure on the bone flap as revealed by scanning electron microscopy (SEM) [9]. Our initial study describing the model identified an important role for MyD88-dependent pathways in bacterial containment and pro-inflammatory mediator production; however, the receptors involved were not identified. MyD88 is an adaptor protein that mediates signaling through Toll-like receptors (with the exception of TLR3), IL-1R, IL-18R, and IL-33R, which culminates in nuclear factor-kB (NF-κB) and mitogen-activated protein kinase (MAPK) activation and the transcriptional activation of a wide array of pro-inflammatory genes [10]. TLRs recognize pathogen-associated molecular patterns (PAMPs) that are conserved across broad groups of microorganisms. *S. aureus* can engage multiple TLRs, including TLR2 and TLR9, which recognize bacterial lipoproteins and non-methylated cytosine-phosphate-guanine (CpG) DNA motifs, respectively [10]. Although both TLR2 and TLR9 are important for *S. aureus* recognition in the setting of infections that are transient and display a more planktonic growth state (i.e., individual bacterial cells causing sepsis or abscesses) [11, 12], nothing is known about the role of either receptor during chronic central nervous system (CNS) biofilm infection. Therefore, to identify the critical receptors upstream of MyD88, we investigated craniotomy infection in TLR2 and TLR9 knockout (KO) mice.

TLR2 and IL-1R signaling are linked by their shared use of MyD88, and since our prior study demonstrated significantly reduced IL-1β levels in MyD88 KO mice during *S. aureus* craniotomy infection [9], this led us to also explore the involvement of caspase-1. Caspase-1 is expressed in an inactive pro-form that undergoes autocatalytic cleavage upon assembly of the inflammasome [13]. The inflammasome is a multi-subunit oligomeric complex composed of a nucleotide oligomerization domain-like receptor (NLR) sensor and apoptosis-associated speck-like protein containing a carboxy-terminal CARD (ASC), which forms a bridge between the majority of NLRs and pro-caspase-1 via homotypic caspase activation and recruitment domain (CARD)-CARD interactions [13–15]. Since all of the known NLRs converge on caspase-1, we also investigated the functional role of caspase-1 during *S. aureus* craniotomy infection using caspase-1 KO animals. Further justification for examining TLR2 and caspase-1 is the known cooperation of both molecules in eliciting IL-1β production [13, 14], although this has not yet been explored in the setting of a chronic CNS biofilm infection.

Our findings identified a critical role for both TLR2 and caspase-1 in preventing bacterial outgrowth in the brain, galea, and bone flap during *S. aureus* craniotomy infection, suggestive of pathway cooperativity. Impaired bacterial containment in both TLR2 and caspase-1 KO mice was associated with global reductions in pro-inflammatory mediator production. However, despite these differences, no significant alterations in leukocyte influx were observed and the infection remained persistent. A significant role for IL-1β in *S. aureus* containment was confirmed by treating caspase-1 KO mice with IL-1β containing microparticles, which returned the elevated bacterial burden to that of WT animals. Co-culture of microglia or macrophages with myeloid-derived suppressor cells (MDSCs), a critical immune population that inhibits leukocyte pro-inflammatory properties [16–18], revealed a key role for TLR2-dependent signaling in eliciting cytokine/chemokine production. Collectively, these findings establish the importance of TLR2 and caspase-1 signaling in preventing *S. aureus* expansion during craniotomy infection via IL-1β production; however, neither molecule is capable of mounting a protective immune response to clear bacteria from the bone flap, brain, or galea in the WT setting. This reveals the existence of additional as of yet undefined pathways that facilitate biofilm establishment and thwart immune-mediated clearance during craniotomy infection.

## Materials and methods

### Ethics statement

This study was conducted in strict accordance with the recommendations in the Guide for the Care and Use of Laboratory Animals of the National Institutes of Health and complies with the ARRIVE guidelines. The protocol was approved by the Institutional Animal Care and Use Committee of the University of Nebraska Medical Center (UNMC; Approval ID: 16-123-10).

### Mice

Both male and female mice were used throughout this study between the ages of 8–12 weeks. TLR2 and TLR9 KO mice (C57BL/6 background) were originally provided by Dr. Shizuo Akira (Osaka University, Japan). NLRP3, ASC, and caspase-1-deficient/caspase-11-positive (*Casp-1*^*−/−*^*/Casp-11*^*+/+*^) mice were obtained from Genentech (South San Francisco, CA). *Casp-1*^*−/−*^*/Casp-11*^*+/+*^ animals were created by Genentech using *Casp-1*^*−/−*^*/Casp-11*^*−/−*^ mice from The Jackson Laboratory (Bar Harbor, ME; Stock #016621; RRID:IMSR_JAX:016621) using a caspase-11 bacterial artificial chromosome to restore caspase-11 expression [19], since caspase-1 KO mice were recently shown to harbor a second mutation in caspase-11 [20]. In addition, some functions previously attributed to caspase-1 may be executed by caspase-11, such as initiation of pyroptosis, responses to infection, and non-canonical activation of caspase-1 by caspase-11 [20]. Therefore, the caspase-11 mutation was not a confounding factor in our studies. All mouse strains were bred in house at UNMC. When animals were designated for experiments, mice of the same sex were randomized into standard density cages (*n* = 5 animals per cage). Mice were housed in a restricted-access BSL2 room equipped with ventilated microisolator cages and maintained at 21 °C under a 12-h light:12-h dark cycle with ad libitum access to water (Hydropac^TM^; Lab Products, Seaford, DE) and Teklad rodent chow (Harlan, Indianapolis, IN) with Nestlets provided for enrichment.

### Mouse model of *S. aureus* craniotomy-associated biofilm infection

A mouse model of *S. aureus* craniotomy infection was utilized as previously described, where biofilm formation on the bone flap leads to infection persistence in the subcutaneous galea and brain [9]. The biofilm properties of the mouse *S. aureus* craniotomy model have been demonstrated by infection persistence out to 9 months, ultrastructural characteristics by SEM, as well as recalcitrance to systemic antibiotics [9, 21], resulting from the metabolic dormancy of a subpopulaion of biofilm-associated bacteria. Briefly, mice were anesthetized with ketamine/xylazine and an incision was made in the skin opposite to the bone flap. A high-speed pneumatic drill was used to create a bone flap (approximately 3 mm in diameter) with care taken to preserve the integrity of the dura. The excised bone flap was incubated with *S. aureus* strain USA300 LAC13c [22] for 5 min to allow for bacterial adherence, rinsed, and immediately reinserted into the skull, whereupon the skin incision was closed by suturing. *S. aureus* colonizes both surfaces of the bone flap, which was also observed at the ultrastructural level by SEM in our previous study of a bone flap from a patient with confirmed methicillin-resistant *S. aureus* infection, and the mouse model also shares magnetic resonance imaging (MRI) attributes with human disease [9].

### Synthesis of IL-1β microparticles, release kinetics, and in vivo administration

To establish the functional importance of IL-1β during *S. aureus* craniotomy infection, animals received IL-1β containing microparticles to provide a continual source of cytokine. To prepare IL-1β-loaded poly(lactide-co-glycolide) (PLGA) microparticles, 30 mg of PLGA (PDLG 5010 PURASORB®) was dissolved in 1 ml chloroform (Millipore-Sigma; Burlington, MA). Next, 12 μg of recombinant mouse IL-1β (BioLegend, San Diego, CA) and 75 μg of bovine serum albumin (BSA, Thermo Fisher, Waltham, MA) in 75 μl of phosphate-buffered saline (PBS) were added to the PLGA solution and sonicated twice at 50% amplitude on ice for a total of 1 min using a sonicator (Qsonica, Newtown, CT) to form the primary emulsion. Next, 2 ml of 1% (w/v) polyvinyl alcohol (PVA, Mw 13000–23000, Millipore-Sigma) dissolved in water was transferred to the primary emulsion and the mixture was sonicated again at 50% amplitude on ice for 30 s to produce the water-in-oil-in-water (W/O/W) emulsion. This emulsion was added dropwise to 40 ml of 0.1% (w/v) PVA in PBS and stirred at 200 rpm at room temperature for 18 h. The microparticles were collected by centrifugation, lyophilized, and stored at – 20 °C before use. The supernatant following microparticle centrifugation was collected to estimate the loading efficiency of IL-1β. The average diameter of microparticles was approximately 0.9 μm as determined by SEM (Additional file 1 A). Control microparticles were prepared using the same protocol with only BSA loaded.

The kinetics of IL-1β release from microparticles in vitro was monitored by ELISA. Briefly, IL-1β-loaded PLGA microparticles (~ 1.8 mg) were suspended in 1 ml of PBS in an Eppendorf tube and incubated at 37 °C for up to 28 days. The releasate was collected at 1 h or days 1, 2, 3, 5, 7, 14, 21, and 28 (Additional file 1 B). At each time point, the microparticles were centrifuged, supernatants collected, and re-suspended in 1 ml of fresh PBS. All releasates were stored at – 80 °C before use. The theoretical loading ratio was determined as ~ 340 ng IL-1β/mg microparticles.

For in vivo experiments, a total dose of 500 ng of IL-1β was administered to each mouse. Based on the estimated loading of IL-1β microparticles, this dose was achieved by mixing 0.75 mg of PLGA microparticles in 7.5 μl of PBS (containing approximately 250 ng encapsulated IL-1β) with 2.5 μl of free IL-1β (BioLegend, 0.2 μg/μl, 250 ng in total), which resulted in a 10-μl solution that contained both free and encapsulated IL-1β. The free IL-1β provided an initial fast release of cytokine, while the PLGA microparticles provided long-term IL-1β release. A total of 5 μl of microparticles were applied at both the ventral and dorsal aspects of the bone flap at the time of infection (~ 250 ng loading dose per location). This approach ensured that both surfaces of the bone flap were bathed in IL-1β to stimulate anti-microbial activity. Mice were sacrificed at day 14 post-infection to quantify IL-1β effects on bacterial burden and leukocyte infiltrates in the galea and brain.

### Tissue collection and processing for bacterial quantification

At the indicated time points post-infection, mice were sacrificed using an overdose of inhaled isoflurane and transcardially perfused with PBS. The bone flap was removed first, followed by the galea, which represents the subcutaneous tissue and associated purulent exudate. Next, the ipsilateral brain hemisphere associated with the infected bone flap was removed and placed in PBS. The bone flap was vortexed in PBS for 30 s followed by a 5-min sonication to dislodge biofilm-associated bacteria. The galea was dissociated in PBS using the blunt end of a plunger from a 3-cc syringe, and the brain was homogenized by pressing through a 70-μm cell strainer and rinsed with PBS. Once all tissues were processed, aliquots were removed to quantify bacterial titers. Titers were determined by serial dilutions on tryptic soy agar (TSA) plates supplemented with 5% sheep blood (Remel, Lenexa, KS) and are expressed as colony-forming units (CFUs). The contralateral hemisphere was also examined; however, the degree of bacterial dissemination was low and not reported.

### Flow cytometry

Following the removal of aliquots from tissue homogenates for quantifying *S. aureus* burden, leukocyte infiltrates in the galea and brain were assessed by flow cytometry. Briefly, brain homogenates were incubated in HBSS containing collagenase IV and DNase I (both from Sigma-Aldrich) for 20 min at 37 ^o^C, whereupon enzymatic activity was inactivated with the addition of 20% fetal bovine serum (FBS). Cells were layered over a 25% Percoll gradient (GE Healthcare, Marlborough, MA) containing 3% FBS and centrifuged at 520×*g* for 20 min with no brake [23]. The upper myelin layer down to the pellet was discarded, and the pellet was resuspended in PBS and filtered to remove remaining particulate material. Galea samples were also centrifuged and filtered, and cells from both the galea and brain were incubated with TruStain fcX (BioLegend) to block non-specific antibody binding and stained with CD11b-FITC, CD45-APC, Ly6G-PE, Ly6C-PerCP-Cy5.5, and F4/80-PE-Cy7 (BioLegend and BD Biosciences, San Diego, CA). Dead cells were excluded using a Live/Dead Fixable Cell Stain Kit (Invitrogen), and analysis was performed using BD FACSDiva software. Myeloid-derived suppressor cells (MDSCs) were classified as CD11b^high^Ly6C^+^Ly6G^+^F4/80^−^, neutrophils as CD11b^low^Ly6C^+^Ly6G^+^F4/80^−^, and monocytes Ly6C^+^Ly6G^*−*^CD11b^+^F4/80^−^ [24] with results presented as the number of events from live, CD45^+^ leukocytes.

### Quantification of inflammatory mediator expression

After mice were sacrificed, the subcutaneous galea and associated purulent exudate were collected with sterile curved forceps along with the ipsilateral hemisphere of the infected brain and both were immediately placed in 500 μl of ice-cold PBS supplemented with a protease inhibitor cocktail tablet (ThermoFisher). The galea was dissociated using the blunt end of a plunger from a 3-cc syringe, and the brain was homogenized by pressing through a 70-μm cell strainer. After processing, samples were centrifuged at 20,817×*g* at 4 °C for 10 min, whereupon cell-free supernatants were collected and stored at − 80 °C until analysis. Inflammatory mediator expression in the brain and galea was quantified using multi-analyte bead arrays (Cat. #MCYTMAG-70 K-PX32; Milliplex, MilliporeSigma) that measured the following molecules: granulocyte colony-stimulating factor (G-CSF), granulocyte-macrophage colony-stimulating factor (GM-CSF), interferon-gamma (IFN-γ), tumor necrosis factor-alpha (TNF-α), interleukin-1 alpha (IL-1α), IL-1β, IL-2, IL-3, IL-4, IL-5, IL-6, IL-7, IL-9, IL-10, IL-12p40, IL-12p70, IL-13, IL-15, IL-17, CXCL10/interferon-inducible protein 10 kDa (IP-10), CXCL1/keratinocyte chemokine (KC), leukemia inhibitory factor (LIF), CXCL5/granulocyte chemotactic protein-2 (LIX), CCL2/macrophage chemoattractant protein-1 (MCP-1), macrophage colony-stimulating factor (M-CSF), CXCL9/monokine induced by IFN-γ (MIG), CCL3/macrophage inflammatory protein-1 alpha (MIP-1α), CCL4/macrophage inflammatory protein-1 beta (MIP-1β), CXLC2/macrophage inflammatory protein-2 (MIP-2), CCL5/regulated upon activated T cell expressed and secreted (RANTES), CCL11 (eotaxin), and vascular endothelial growth factor (VEGF). Results were normalized to the amount of protein recovered from tissues to correct for differences in sampling size as well as titers to account for disparities in bacterial burden between the various KO strains and WT mice.

### Hematoxylin and eosin (H&E) staining

TLR2 KO, caspase-1 KO, and WT animals were deeply anesthetized using sodium pentobarbital and transcardially perfused with PBS followed by 4% paraformaldehyde (PFA). To preserve the inflammatory response in the galea, the entire head was collected and post-fixed in 4% PFA for 4 days. The head was decalcified in 20% ethylenediaminetetraacetic acid (EDTA) for 14 days and cryoprotected with 30% sucrose for 4 days prior to embedding in optimal cutting temperature medium. Cryostat sections (16 μm) were collected every 100 μm and mounted on SuperFrost slides (ThermoFisher), and representative tissue sections encompassing the craniotomy infection site were rehydrated and subjected to H&E staining. Stained sections were imaged on a Ventana iScan HT scanner (Roche, Indianapolis, IN) and are presented at × 2 magnification to demonstrate the extent of infection involvement.

### Preparation of primary mouse microglia, macrophages, and MDSCs for co-culture studies

Primary microglia were prepared from 1–2-day-old WT, TLR2 KO, and caspase-1 KO pups as previously described with modifications [25]. Briefly, pups were euthanized using an overdose of inhaled isoflurane and the cortex was removed and minced in ice-cold PBS using sterile forceps. The tissue slurry was incubated for 20 min at 37 °C with 0.5% trypsin-EDTA to create a single-cell suspension. Trypsin was inactivated with culture medium, and cells were centrifuged, passed through a 70-μm filter to remove cell aggregates, and resuspended in culture medium [Dulbecco’s modified Eagle medium (DMEM) 4.5 g/L glucose supplemented with 10% FBS, penicillin/streptomycin/fungizone, OPI medium supplement (oxalacetic acid, pyruvate, insulin; Millipore-Sigma), and 0.5 ng/ml recombinant mouse GM-CSF (BioLegend)] and added to 75 cm^2^ flasks. The culture medium was replaced after 4 days to remove non-adherent cells, and after another 4 days, cells were trypsinized and transferred to 175 cm^2^ flasks. When mixed glial cultures reached confluence (typically between 10 and 14 days), microglia were detached from the astrocyte monolayer by tapping the flask by the hand. Prior to each experiment, the purity of recovered microglia was assessed by flow cytometry using CD11b-FITC (BioLegend) and was routinely greater than 95%. Bone marrow-derived macrophages and MDSCs were prepared from the bone marrow of adult WT, TLR2 KO, and caspase-1 KO mice (8–12 weeks of age) as previously described [26, 27]. Briefly, the bone marrow was passed through a 70-μm filter and red blood cells (RBCs) were eliminated using a RBC lysis buffer (BioLegend). Macrophages were expanded over 7 days in RPMI-1640 containing 10% FBS, penicillin/streptomycin/fungizone and 5% conditioned medium from L929 fibroblasts as a source of M-CSF at 37 °C, 5% CO_2_. FACS analysis revealed that > 98% of cells were macrophages, based on CD11b and F4/80 staining. For MDSCs, bone marrow cells were incubated for 4 days in RPMI-1640 supplemented with 10% FBS, GM-CSF (40 ng/ml), and G-CSF (40 ng/ml) at 37 °C, 5% CO_2_ with IL-6 (40 ng/ml) added at day 3. MDSCs were purified from the mixed cell population using Ly6G magnetic beads (Miltenyi Biotec, San Diego, CA), which resulted in a cell purity of 95% and were confirmed for co-expression of Ly6G and Ly6C [27].

For co-culture experiments, microglia and macrophages were plated at 5 × 10^4^/well in a 96-well plate overnight, whereupon an equal number of MDSCs were added the following day. Cells were stimulated with 10 ng/ml of the synthetic TLR2 triacylated lipoprotein agonist Pam3CysSerLys4 (Pam3CSK4, Invivogen, San Diego, CA) for 24 h, and conditioned medium was collected for quantification of IL-1β, G-CSF (DuoSet, R&D Systems, Minneapolis, MN), and CCL2 (BioLegend) by enzyme-linked immunosorbent assay (ELISA). The level of sensitivity for ELISAs was 3.2 pg/ml.

### Gentamicin protection assay

WT, TLR2 KO, and caspase-1 KO microglia or macrophages were plated at 5 × 10^4^/well in a 96-well plate overnight, whereupon cells were exposed to live *S. aureus* at a multiplicity of infection (MOI) of 10:1 (bacteria to macrophage) for 1 h. Next, cells were washed and treated with medium containing 100 μg/ml gentamicin for 30 min to kill remaining extracellular bacteria, whereupon the gentamicin concentration was reduced to 1 μg/ml. Microglia and macrophages were lysed with water at 0, 2, 4, 6, and 24 h after low-dose gentamicin treatment to quantify the number of surviving intracellular bacteria.

### Statistics

Significant differences between groups were determined using an unpaired Student’s *t* test or a one-way ANOVA with Tukey’s multiple comparison test using GraphPad Prism version 6 (La Jolla, CA) where a *p* value < 0.05 was considered statistically significant.

## Results

### TLR2, but not TLR9, is critical for bacterial containment during *S. aureus* craniotomy infection

**Fig. 1:**
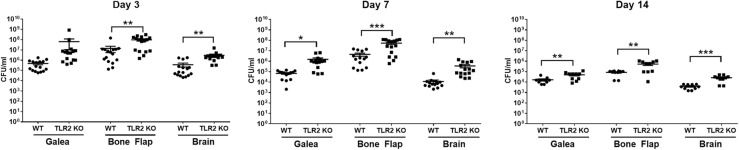
TLR2 is important for *S. aureus* containment during craniotomy infection. WT and TLR2 KO mice were sacrificed at days 3, 7, or 14 following *S. aureus* craniotomy infection, whereupon bacterial burden in the galea, bone flap, and brain was quantified. Results were combined from 3 independent experiments (*n* = 10–16 mice/group) and analyzed by unpaired Student’s *t* test (*p < 0.05, **p < 0.01, ***p < 0.001)

Infectious complications after craniotomy are typically not able to be resolved without performing surgery to debride the infected bone flap and surrounding purulent material [28]. This is because bacteria adherent to the bone flap have formed a biofilm, which is recalcitrant to antibiotics and immune-mediated clearance [4, 29]. Once the biofilm has been disrupted, by aggressive disinfection of the bone flap intra-operatively or removal if the bone flap cannot be salvaged, the remaining bacteria are antibiotic susceptible, since they become metabolically active. Our recent reports established the biofilm properties associated with the mouse model of *S. aureus* craniotomy infection, including biofilms that were ultrastructurally indistinguishable from an infected bone flap of a patient with confirmed *S. aureus* infection as demonstrated by SEM [9] and the failure to clear infection in the mouse craniotomy model following treatment with systemic antibiotics, which is the functional definition of a biofilm based on its antibiotic tolerance [21]. However, our original study revealed that mice deficient in MyD88, the adaptor molecule responsible for TLR, IL-1R, IL-18R, and IL-33R signaling, was essential for bacterial containment during acute *S. aureus* craniotomy infection [9]. The fact that craniotomy infections persist in the setting of normal MyD88 activity suggests that MyD88-dependent signals contribute to keeping bacterial expansion in check, but are not sufficient for clearing infection. Two TLRs that are pertinent to the gram-positive pathogen *S. aureus* are TLR2 and TLR9 that recognize bacterial lipoproteins and non-methylated CpG DNA motifs, respectively [10]. Therefore, we examined the functional importance of both receptors in bacterial containment during *S. aureus* craniotomy infection using TLR2 and TLR9 KO mice. TLR2-dependent signaling was critical for controlling *S. aureus* burden in the brain and bone flap as early as day 3 post-infection, with titers becoming significantly elevated in the galea at day 7 (Fig. 1). Bacterial counts remained elevated at day 14 post-infection, whereupon a significant number of bone flaps were extruded by day 21 in TLR2 KO mice (~ 40%, Table 1), which was attributed to erosion of the overlying skin resulting from unchecked bacterial replication. In contrast, although TLR9 is also capable of triggering pro-inflammatory responses, it had no significant impact on bacterial burden, although titers trended higher in the brains of TLR9 KO mice (Additional file 2).

**Table 1:** Loss of bone flaps in TLR2 and caspase-1 KO mice at later stages of infection

Mouse strain	Bone flaps lost—day 21	Bone flaps lost—day 28
Wild type	0/16	0/16
TLR2 KO	6/16	7/16
Caspase-1 KO	3/16	6/16

**Fig. 2:**
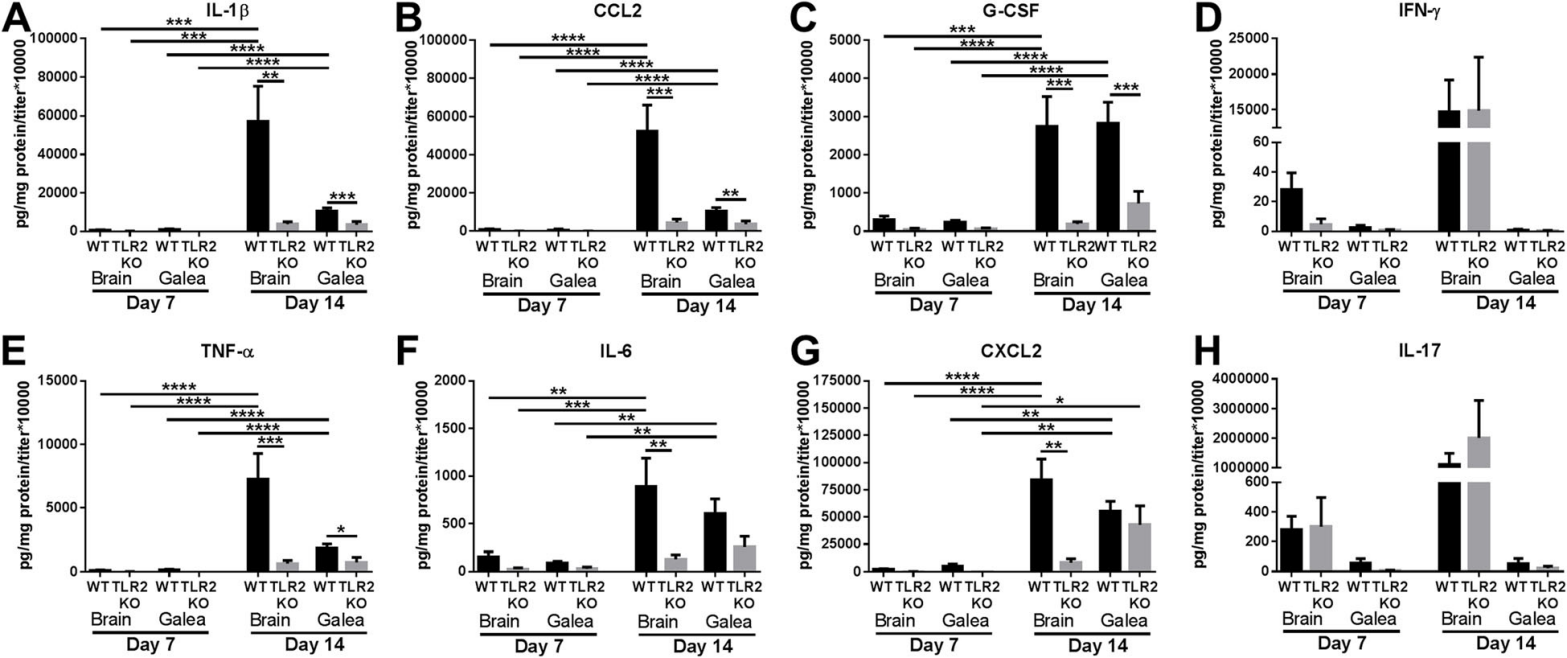
Pro-inflammatory mediator production during *S. aureus* craniotomy infection is primarily TLR2-dependent. WT and TLR2 KO mice were sacrificed at days 7 or 14 following *S. aureus* craniotomy infection, whereupon (**a**) IL-1β, (**b**) CCL2, (**c**) G-CSF, (**d**) IFN-γ, (**e**) TNF-α, (**f**) IL-6, (**g**) CXCL2, and (**h**) IL-17 production was quantified using Milliplex multi-analyte bead arrays. Results were combined from two independent experiments (*n* = 5–10 mice/group) and analyzed by one-way ANOVA with Tukey’s multiple comparison test (*p < 0.05, **p < 0.01, ***p < 0.001, ****p < 0.0001)

TLR2 activation induces the robust expression of numerous pro-inflammatory mediators via NF-κB and MAPK pathways [30]. To determine whether the heightened bacterial burden in TLR2 KO mice could be attributed to impaired pro-inflammatory activity, cytokines/chemokines were quantified in the brain and galea of TLR2 KO and WT animals. The inflammatory mediators selected represent a broad range of pro-inflammatory cytokines/chemokines whose expression has been shown to be TLR2-dependent [10, 30]. When normalized to bacterial titer to account for the expansion of *S. aureus* in TLR2 KO animals, the expression of several inflammatory mediators was significantly reduced, including IL-1β, CCL2, G-CSF, TNF-α, IL-6, and CXCL2, whereas IFN-γ and IL-17 were unchanged (Fig. 2). An interesting finding was that some mediators were more affected by TLR2 loss in the brain compared to the galea, including IL-1β, CCL2, TNF-α, IL-6, and CXCL2, which was particularly evident at day 14 post-infection. In contrast, G-CSF was equally affected by TLR2 deletion in both the galea and brain (Fig. 2). Collectively, these findings suggest that the failure to contain *S. aureus* burden in the brain and galea during craniotomy infection in TLR2 KO mice may result from the inability to elicit maximal pro-inflammatory mediator production. An enigma that remains is that although these pro-inflammatory factors are elicited in WT animals, they are still not sufficient to clear biofilm infection.

**Fig. 3:**
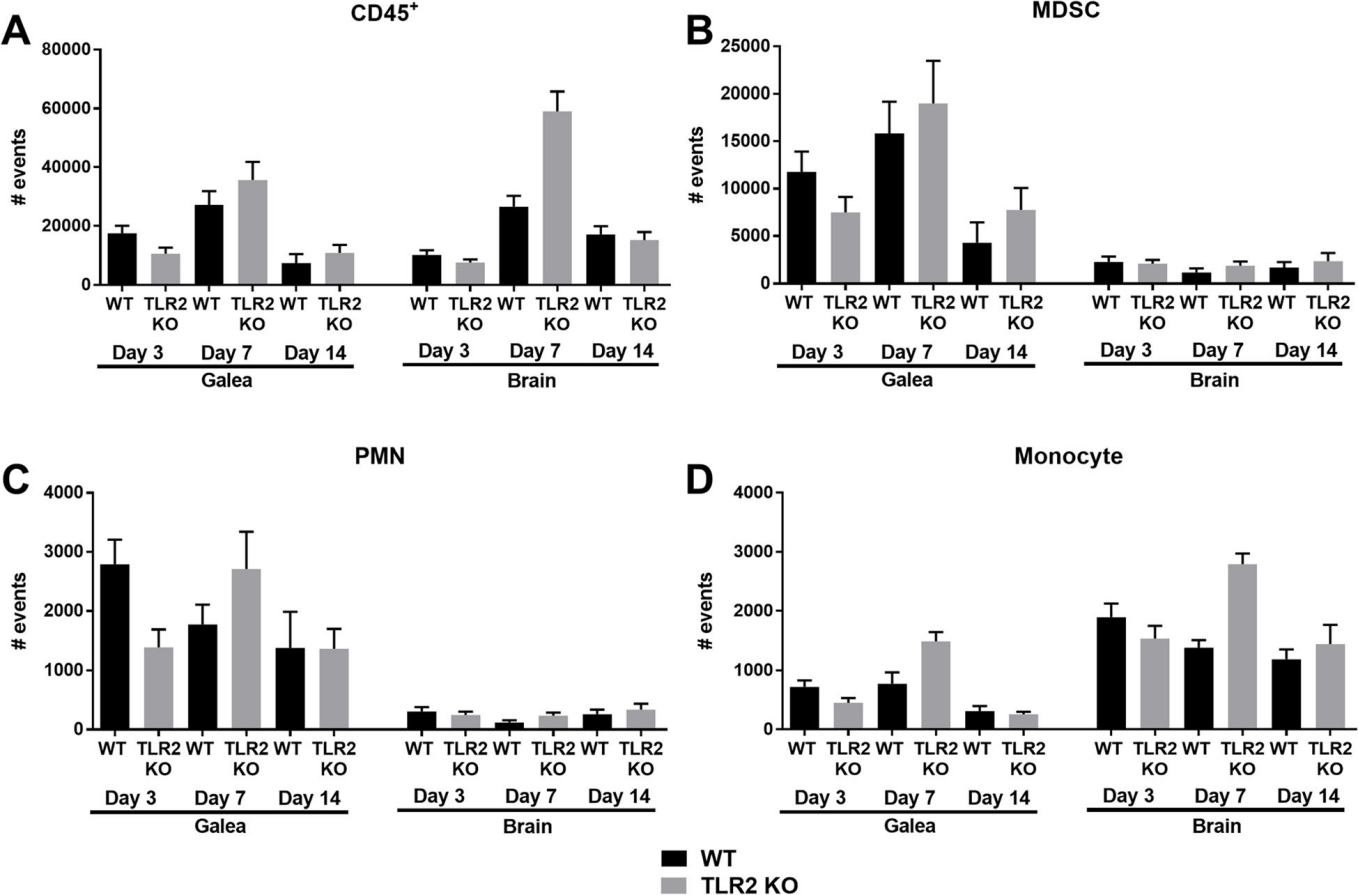
TLR2 does not regulate leukocyte influx during *S. aureus* craniotomy infection. WT and TLR2 KO mice were sacrificed at days 3, 7, or 14 following *S. aureus* craniotomy infection, whereupon (**a**) total CD45^+^ leukocyte, (**b**) MDSC (CD11b^high^Ly6C^+^Ly6G^+^F4/80^−^), (**c**) neutrophil (CD11b^low^Ly6C^+^Ly6G^+^F4/80^*−*^), and (**d**) monocyte (Ly6C^+^Ly6G^*−*^CD11b^+^F4/80^*−*^) infiltrates were quantified in the galea and brain. Results were combined from 3 independent experiments (*n* = 10–16 mice/group)

Given the reduction in CXCL2 and CCL2 expression in TLR2 KO mice, key chemoattractants for MDSCs and neutrophils versus monocytes, respectively [31–33], we next determined whether this would translate into impaired leukocyte recruitment. Surprisingly, no significant differences in leukocyte influx were observed in the galea or brain of TLR2 KO and WT mice at any of the time points examined (Fig. 3) despite the fact that the former displayed significantly higher bacterial burdens. Although some fluctuations in leukocyte infiltrates occurred in the brain and galea of TLR2 KO mice at days 3 and 7 post-infection, these did not reach statistical significance (Fig. 3). Collectively, the lack of overt changes in leukocyte recruitment in TLR2 KO animals suggests the existence of redundant chemotactic signals that are elicited in a TLR2-independent manner to promote leukocyte influx into the brain and galea during *S. aureus* craniotomy infection.

**Fig. 4:**
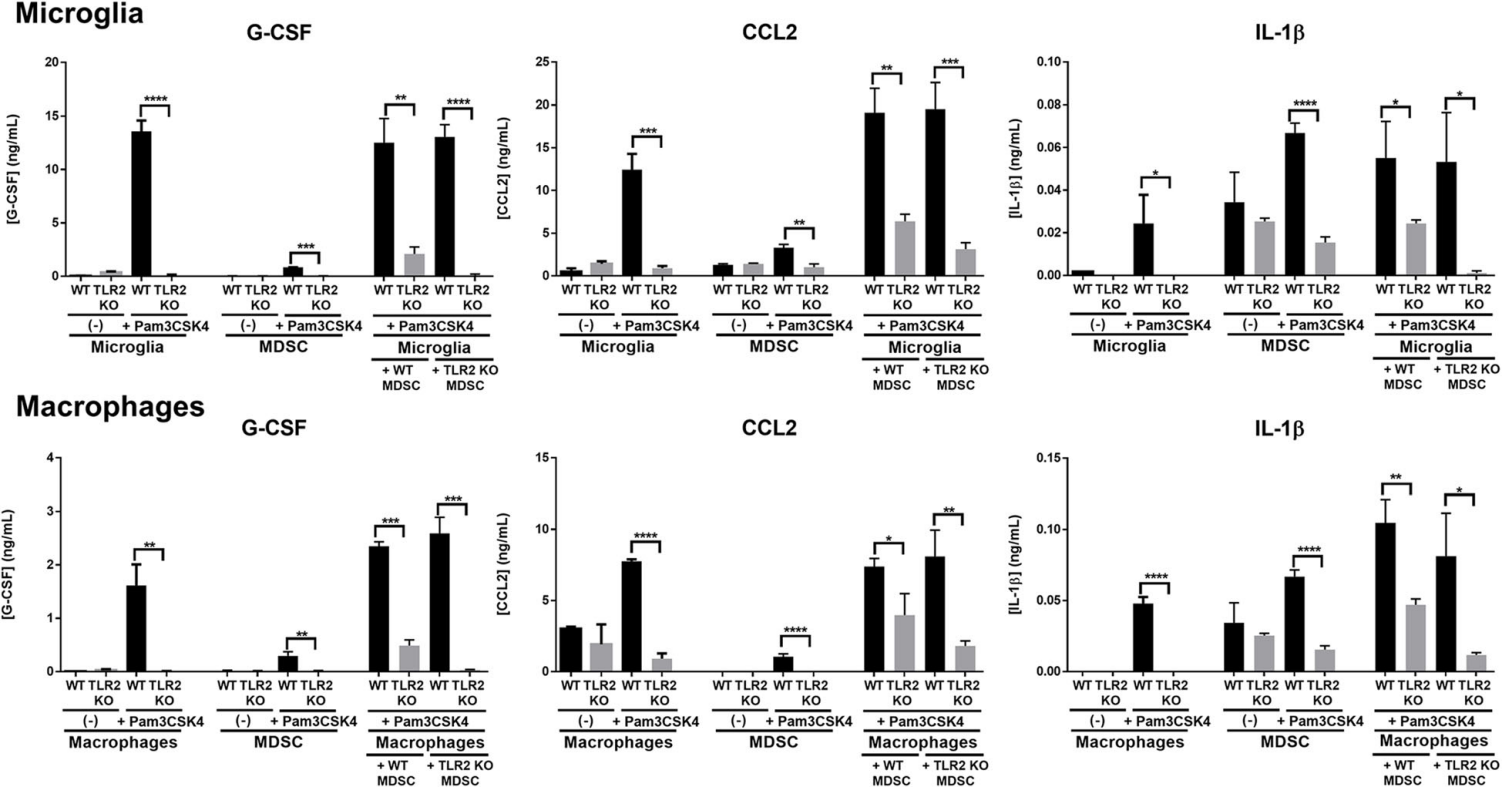
TLR2-dependent signaling is critical for eliciting inflammatory mediator release during MDSC co-culture with microglia or macrophages. Primary microglia or bone marrow-derived macrophages were cultured alone or with bone marrow-derived MDSCs in the presence/absence of the synthetic TLR2 ligand Pam3CSK4 (10 ng/ml) for 24 h, whereupon G-CSF, CCL2, and IL-1β production was measured by ELISA. Results are from one experiment (*n* = 3 biological replicates/treatment) that was independently replicated four times and analyzed by unpaired Student’s *t* test (*p < 0.05, **p < 0.01, ***p < 0.001, ****p < 0.0001)

MDSCs produce molecules that inhibit macrophage pro-inflammatory activity [16, 34, 35]. During *S. aureus* craniotomy infection, MDSCs are present in both the brain and galea (Fig. 3), where they would encounter microglia and monocytes/macrophages, respectively. To investigate the crosstalk between these cell types and the impact of TLR2 on inflammatory mediator production, MDSC-microglia and MDSC-macrophage co-cultures were treated with the synthetic TLR2 ligand Pam3CSK4, which models *S. aureus* membrane-associated lipoproteins [36]. Co-cultures with TLR2 KO cells produced significantly less G-CSF, CCL2, and IL-1β compared to WT co-cultures (Fig. 4), which mirrored the TLR2-dependent recognition of Pam3CSK4 by microglia, macrophages, and MDSCs alone (Fig. 4). These observations support our in vivo findings that pro-inflammatory mediator expression was significantly reduced in the brain and galea of infected TLR2 KO mice (Fig. 2).

### Caspase-1-dependent signals are critical for bacterial containment during *S. aureus* craniotomy infection

**Fig. 5:**
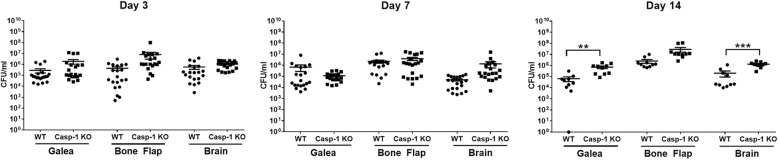
Caspase-1 is important for *S. aureus* containment in the galea and brain during craniotomy infection. WT and caspase-1 (Casp-1) KO mice were sacrificed at days 3, 7, or 14 following *S. aureus* craniotomy infection, whereupon bacterial burden in the galea, brain, and bone flap was quantified. Results were combined from 3 independent experiments (*n* = 10–20 mice/group) and analyzed by unpaired Student’s *t* test (**p < 0.01, ***p < 0.001)

TLR2 and caspase-1 inflammasome signaling are linked, where TLR2 agonists stimulate pro-IL-β production, which requires processing by caspase-1 to secrete the mature cytokine [13, 15]. Since IL-1β expression was significantly reduced in the brain of TLR2 KO mice as well as TLR2-deficient microglia and macrophages, we next examined the functional importance of caspase-1 during *S. aureus* craniotomy infection using caspase-1 KO mice. Importantly, as commercially available caspase-1 KO animals also harbor a deletion in caspase-11 [20], the mice used here were only deficient for caspase-1 because caspase-11 was restored [19]. Similar to our findings with TLR2 KO animals, caspase-1 also contributed to *S. aureus* containment during craniotomy infection but with distinct kinetics. Specifically, heightened bacterial burdens were primarily evident at day 14 post-infection in the galea and brain of caspase-1 KO animals (Fig. 5), whereas TLR2 was critical during acute infection (i.e., day 3) and beyond (Fig. 1).

**Fig. 6:**
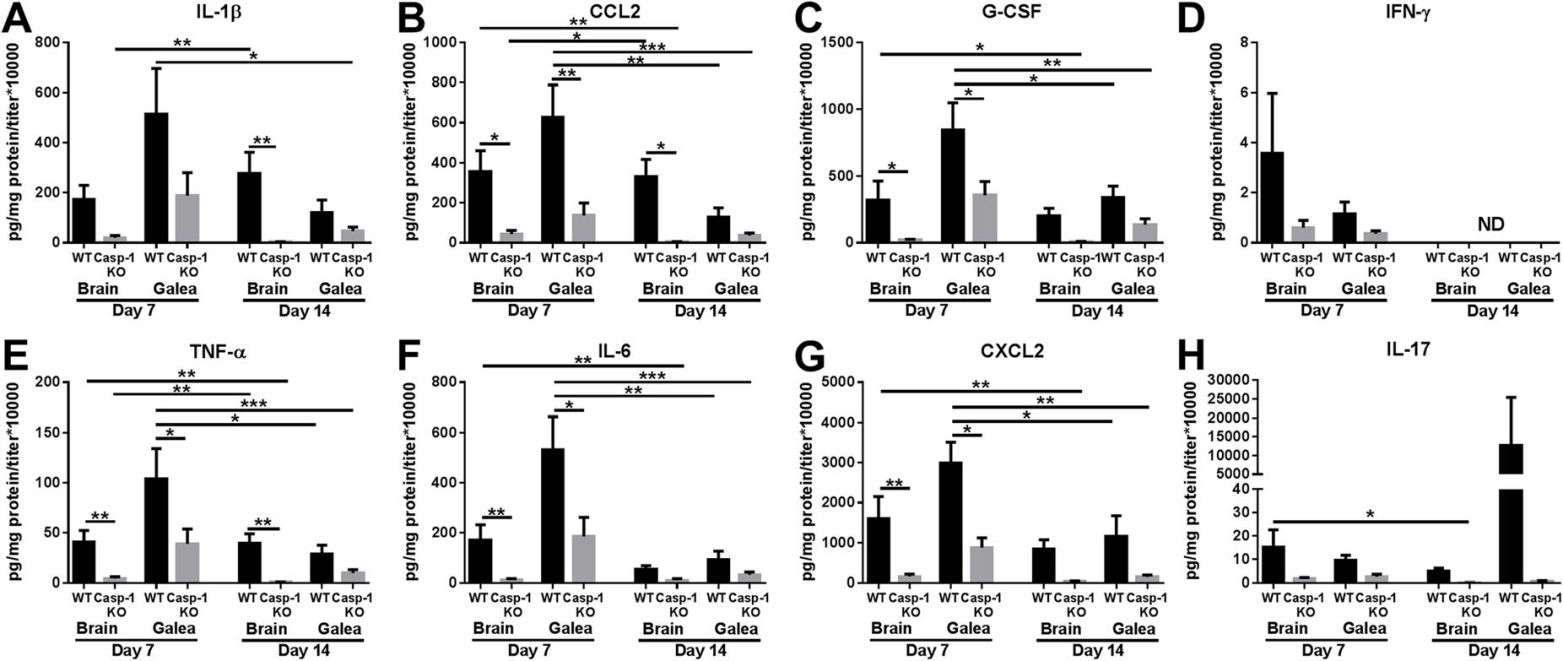
Pro-inflammatory mediator production during *S. aureus* craniotomy infection is influenced by caspase-1 activation. WT and caspase-1 (Casp-1) KO mice were sacrificed at days 7 or 14 following *S. aureus* craniotomy infection, whereupon (**a**) IL-1β, (**b**) CCL2, (**c**) G-CSF, (**d**) IFN-γ, (**e**) TNF-α, (**f**) IL-6, (**g**) CXCL2, and (**h**) IL-17 production was quantified using Milliplex multi-analyte bead arrays. Results were combined from two independent experiments (*n* = 8–10 mice/group) and analyzed by one-way ANOVA with Tukey’s multiple comparison test (*p < 0.05, **p < 0.01, ***p < 0.001). ND not determined

**Fig. 7:**
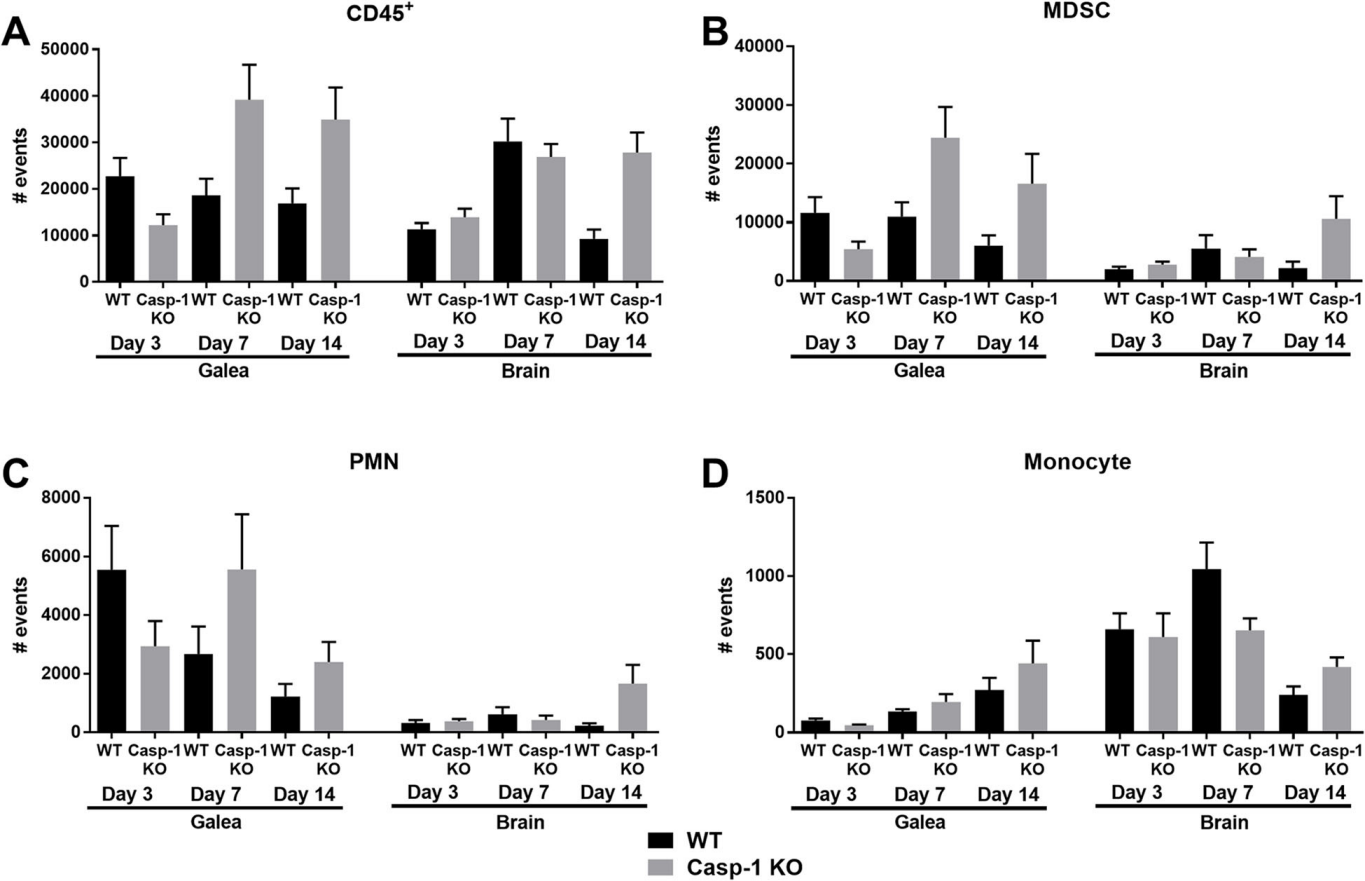
Caspase-1 does not regulate leukocyte influx during *S. aureus* craniotomy infection. WT and caspase-1 (Casp-1) KO mice were sacrificed at days 3, 7, or 14 following *S. aureus* craniotomy infection, whereupon (**a**) total CD45^+^ leukocyte, (**b**) MDSC (CD11b^high^Ly6C^+^Ly6G^+^F4/80^*−*^), (**c**) neutrophil (CD11b^low^Ly6C^+^Ly6G^+^F4/80^*−*^), and (**d**) monocyte (Ly6C^+^Ly6G^*−*^CD11b^+^F4/80^*−*^) infiltrates were quantified in the galea and brain. Results were combined from 3 independent experiments (*n* = 15 mice/group)

We next examined the impact of caspase-1 loss on the inflammatory milieu in the brain and galea during craniotomy infection. Although the infectious burden was not dramatically different at day 7 post-infection, caspase-1 KO mice displayed significant reductions in numerous pro-inflammatory mediators at this interval, including IL-1β (Fig. 6), which preceded the elevation in bacterial burden at day 14 (Fig. 5). Similar to TLR2 KO mice, despite the decrease in pro-inflammatory cytokine and chemokine expression, leukocyte influx into the brain or galea was not significantly different between caspase-1 KO and WT animals, although some fluctuations were observed (Fig. 7). Another similarity between caspase-1 and TLR2 KO mice was the loss of bone flaps at later intervals, where 18% and 38% of caspase-1 KO animals had no bone flaps at days 21 or 28 post-infection, respectively (Table 1).

**Fig. 8:**
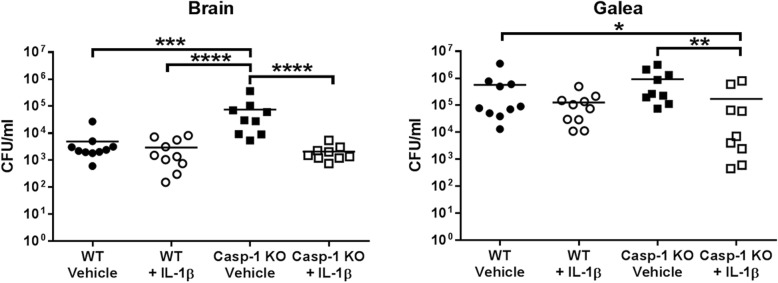
Exogenous IL-1β prevents *S. aureus* outgrowth in caspase-1-deficient mice during craniotomy infection. WT and caspase-1 (Casp-1) KO mice received IL-1β containing or control (vehicle) microparticles at the dorsal and ventral aspects of the bone flap on the day of *S. aureus* infection and were sacrificed at day 14 to quantify bacterial burden in the brain and galea. Results were combined from two independent experiments (*n* = 9–10 mice/group) and analyzed by one-way ANOVA with Tukey’s multiple comparison test (*p < 0.05, **p < 0.01, ***p < 0.001, ****p < 0.0001)

To confirm that the mechanism responsible for exaggerated bacterial burden in caspase-1 KO mice resulted from attenuated IL-1β production, animals received IL-1β containing microparticles at the time of infection to provide a continual source of cytokine. IL-1β microparticles significantly reduced *S. aureus* burden in the brain and galea of caspase-1 KO mice compared to empty microparticles, achieving titers equivalent to WT animals (Fig. 8). In contrast, IL-1β microparticles had no effect on *S. aureus* infection in WT mice (Fig. 8). No significant differences in leukocyte infiltrates were observed in caspase-1 KO mice treated with IL-1β microparticles (data not shown). Bone flaps were lost in mice receiving IL-1β microparticles, which precluded us from making comparisons of bone flap titers across treatment groups. This could have resulted from a heightened pro-inflammatory response elicited by prolonged exogenous IL-1β action, although this remains speculative. These findings establish the importance of IL-1β in containing *S. aureus* expansion during craniotomy infection.

The most characterized inflammasome complex is formed by NLRP3 and the adaptor ASC that bridges NLRP3 to caspase-1 [13, 15]. To investigate whether these inflammasome proteins were responsible for sensing *S. aureus* to trigger caspase-1 activation, craniotomy infection was examined in NLRP3 and ASC KO animals. Interestingly, bacterial burden was similar in the brain and galea of NLRP3 and ASC KO mice compared to WT animals (Additional file 3) with no significant alterations in leukocyte recruitment in either compartment (data not shown), suggesting that neither NLRP3 nor ASC is responsible for caspase-1 activation. Although bacterial burden was significantly different on the bone flap of NLRP3 and ASC KO mice at days 3 and 14 post-infection (Additional file 3), this did not translate into altered disease progression. Collectively, these findings demonstrate the functional importance of caspase-1 in bacterial containment during *S. aureus* craniotomy infection with a NLR sensor that remains to be identified.

**Fig. 9:**
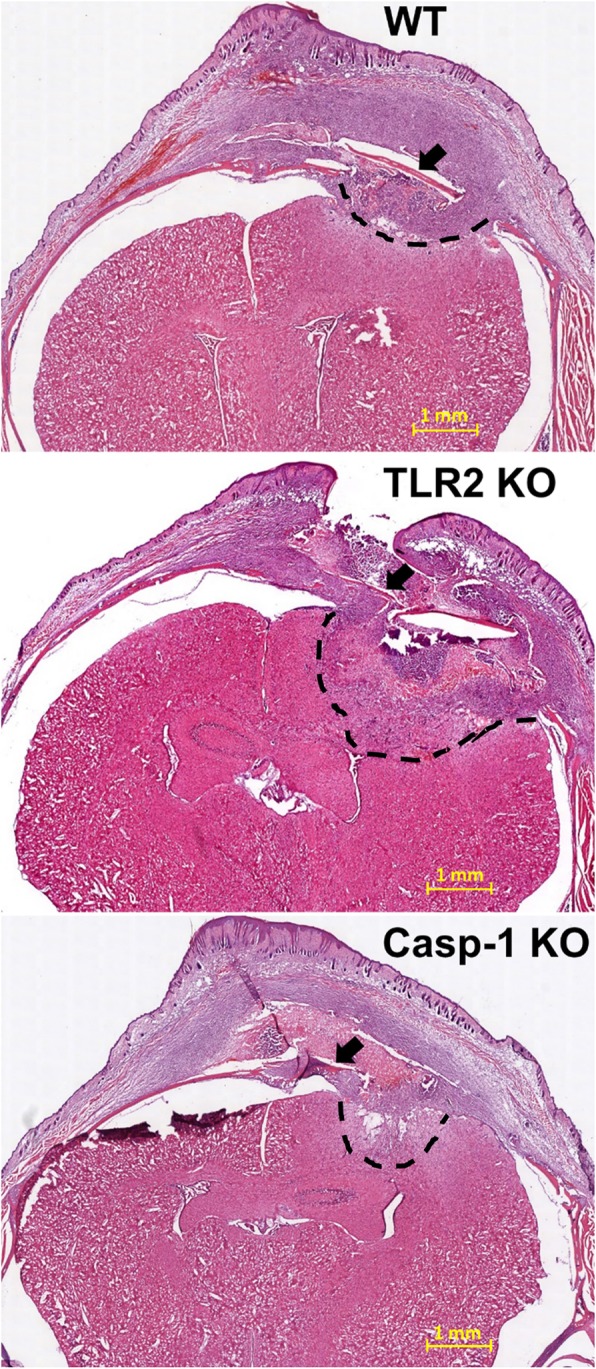
TLR2 limits tissue damage during *S. aureus* craniotomy infection. WT, TLR2 KO, and caspase-1 (Casp-1) KO mice were sacrificed at day 7 following *S. aureus* craniotomy infection, whereupon cryosections of the head were processed for H&E staining to visualize the extent of inflammation in the galea and brain. Images presented are representative of 3 mice/group (× 2 magnification; scale bar = 1 mm). Arrows depict the bone flap, and the extent of brain parenchymal damage is denoted with dashed lines

To investigate the degree of CNS pathology in TLR2 and caspase-1 KO mice during craniotomy infection, histological assessments were performed. H&E staining revealed heightened inflammation following TLR2 loss, which extended beyond the vicinity of the infected bone flap and into the underlying brain parenchyma (Fig. 9). In contrast, inflammatory changes were similar between caspase-1 KO and WT mice. The increased tissue pathology of TLR2 KO versus caspase-1 KO animals agrees with the finding that *S. aureus* burden was increased early post-infection and more dramatically in TLR2 KO mice. This supports a key role for TLR2-dependent effector mechanisms to limit parenchymal damage in the wild type setting.

**Fig. 10:**
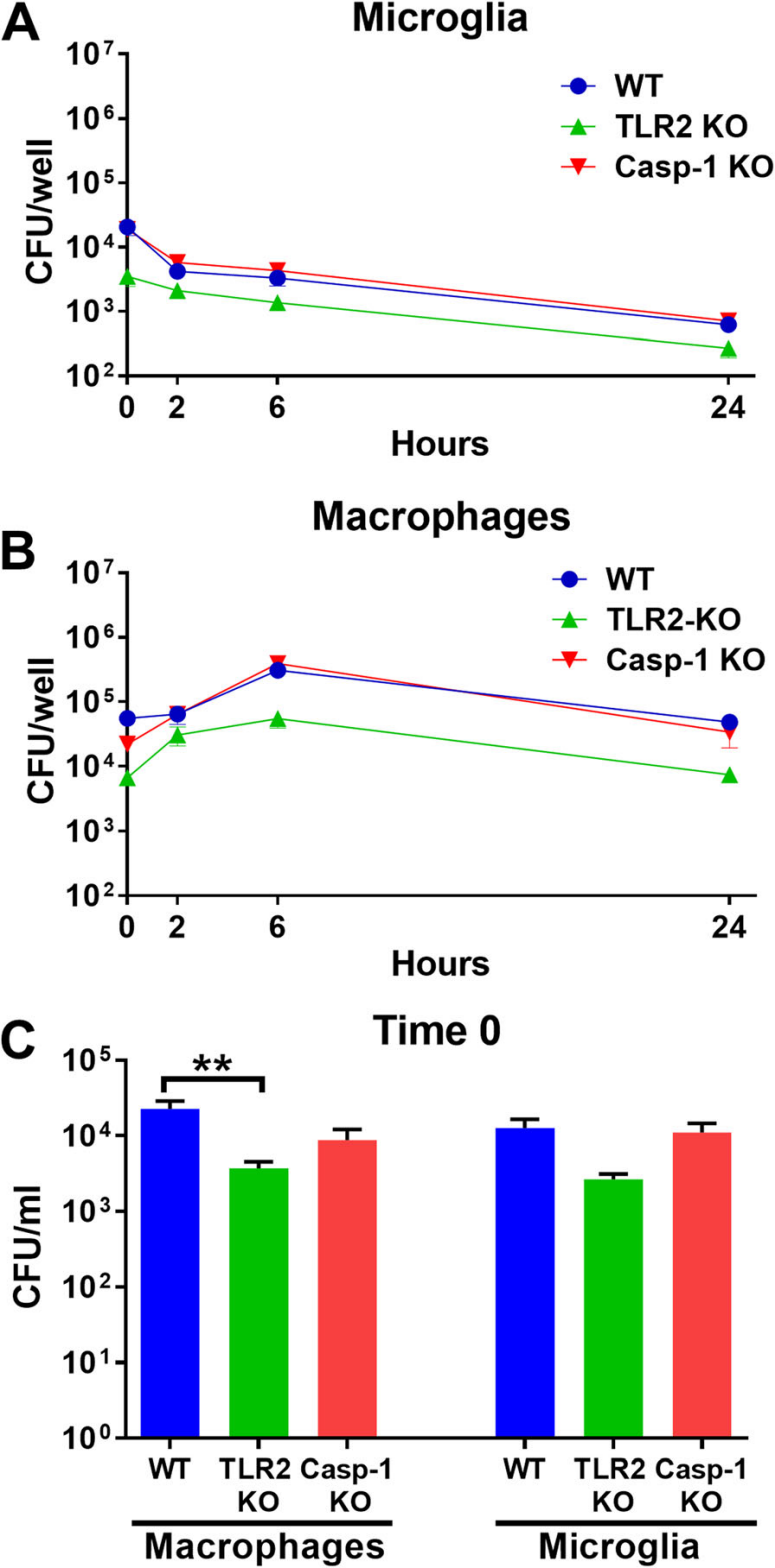
TLR2, but not caspase-1, influences early intracellular survival in microglia and macrophages. Gentamicin protection assays were performed on primary microglia or bone marrow-derived macrophages from WT, TLR2 KO, and caspase-1 (Casp-1) KO mice using a MOI of 10:1 (bacteria to cell). Time course of intracellular bacterial survival in (**a**) microglia and (**b**) macrophages with (**c**) quantification of intracellular burdens at time 0 (immediately following high dose gentamicin treatment). Results are from one experiment (*n* = 4 biological replicates/treatment) that was independently replicated four times and analyzed by one-way ANOVA with Tukey’s multiple comparison test (**p < 0.01)

Finally, we examined whether TLR2 or caspase-1 influenced microglial or macrophage bactericidal activity, which might account for the unchecked replication of *S. aureus* in vivo following the loss of these molecules. Gentamicin protection assays revealed that intracellular bacterial burden was reduced in both TLR2 KO microglia and macrophages immediately following high-dose gentamicin treatment (time 0), whereas caspase-1 KO cells were equivalent to WT (Fig. 10). The reason for reduced intracellular *S. aureus* in TLR2 KO microglia and macrophages is unclear, since TLR2 is not a phagocytic receptor [37]; however, TLR2-dependent signaling is known to augment phagocytic receptor expression [38], which may be responsible for this finding. Nevertheless, intracellular bacteria were reduced in TLR2 KO cells at 24 h (Fig. 10a, b), revealing the existence of TLR2-independent mechanisms of bactericidal activity.

## Discussion

**Fig. 11:**
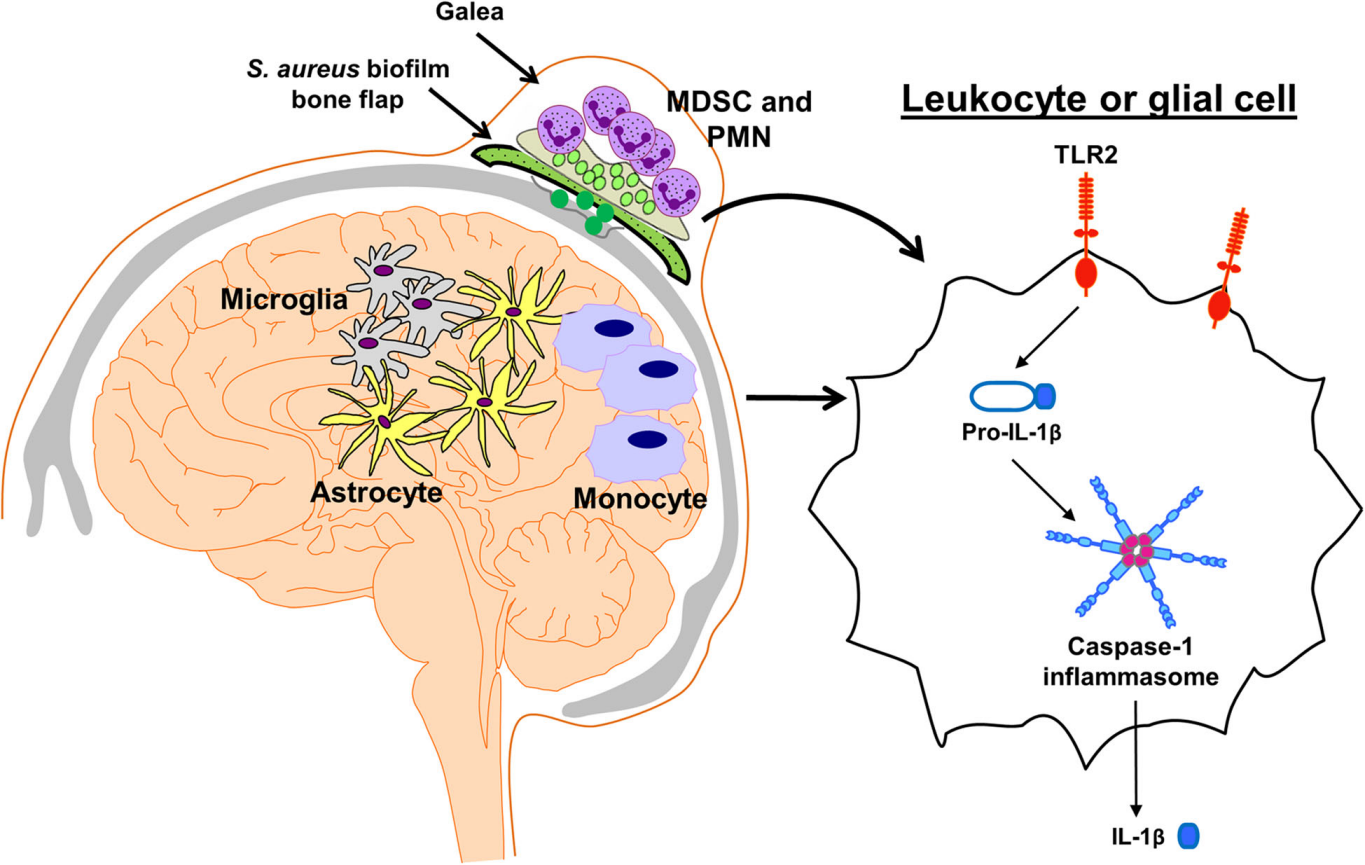
Interplay between TLR2 and caspase-1 signaling during *S. aureus* craniotomy infection. *S. aureus* craniotomy infection is typified by biofilm formation on the bone flap, which results in an inflammatory response in the galea that is dominated by myeloid-derived suppressor cell (MDSC) and neutrophil (PMN) infiltrates, whereas monocytes are more numerous in the inflamed brain. *S. aureus* triggers TLR2-dependent signaling, which leads to pro-IL-1β production that requires activation by the caspase-1 inflammasome for processing to the mature cytokine. IL-1β is critical for *S. aureus* containment during craniotomy infection, but is not sufficient for bacterial clearance, since biofilm infections persist in an immune competent host

Our prior report demonstrated a critical role for MyD88-dependent signaling during *S. aureus* craniotomy infection, where MyD88 KO mice displayed heightened bacterial burden that translated into significant morbidity [9]. In the current study, we demonstrate that the MyD88-dependent receptor TLR2 is critical for *S. aureus* containment in the brain and galea during craniotomy infection. Likewise, our data also support an important role for IL-1β-dependent signaling, presumably via the IL-1R that also requires the MyD88 adaptor, since caspase-1 KO mice produced significantly less IL-1β, which translated into the failure to limit *S. aureus* replication in the brain and galea. This was confirmed by providing exogenous IL-1β to caspase-1 KO animals via microparticle delivery, which reduced bacterial burden to levels observed in WT mice. The relationship between TLR2 and caspase-1 is also supported by the disparate kinetics of each KO mouse during infection. For example, TLR2 was important during acute infection, a period where initial bacterial recognition via TLR2 is essential for promoting maximal pro-inflammatory activity and as a consequence, pro-IL-1β production [15]. In contrast, bacterial burden was most affected in caspase-1 KO mice at day 14 post-infection, in agreement with caspase-1 activity occurring subsequent to TLR2 signaling to maximize IL-1β production (Fig. 11) [14].

Although *S. aureus* burden was not dramatically affected at day 7 post-infection in caspase-1 KO mice, significant reductions in numerous pro-inflammatory mediators were observed at this interval, which preceded the elevation in bacterial burden at day 14. This finding suggests a critical role for IL-1β signaling in promoting pro-inflammatory mediator production during craniotomy infection. Specifically, TLR2 activation by *S. aureus*-derived PAMPs triggers pro-IL-1β production that is processed via caspase-1 cleavage (Fig. 11). Upon release, IL-1β binds to the IL-1R to amplify pro-inflammatory mediator release, effectively perpetuating the anti-bacterial response. The loss of either TLR2 or caspase-1 dramatically attenuated cytokine/chemokine production during *S. aureus* craniotomy infection, which supports the lack of this positive feedback loop and the inability to contain bacterial growth. In addition, these findings are supported by our prior study in MyD88 KO mice [9], where animals were exquisitely sensitive to craniotomy infection, since MyD88 is required for both TLR2 and IL-1R signaling, revealing the consequences of negating redundant bacterial recognition pathways. This is substantiated by the finding that neither TLR2 nor caspase-1 KO mice displayed overt signs of morbidity and survived the infection similar to WT animals. Of note, caspase-1 has been shown to cleave over 500 other proteins besides pro-IL-1β [39, 40] that may also exert protective effects in limiting *S. aureus* outgrowth in a WT setting. For example, caspase-1 has been implicated in mitochondrial damage, cleavage of glycolytic enzymes, degradation of proteins involved in immune sensing, and release of proteins that do not contain a signal sequence for secretion [41]. Therefore, caspase-1 could influence additional pathology during *S. aureus* craniotomy infection beyond its role in pro-IL-1β processing; however, this possibility remains speculative.

Our findings provide important insights into biofilm regulation during craniotomy infection. First, the fact that TLR2 and caspase-1 deficiency results in bacterial outgrowth demonstrates that innate immune recognition plays a key role in limiting bacterial expansion in the WT setting. Of note, the effects of TLR2 and caspase-1 on *S. aureus* burden were most evident in the brain and galea, regions that are unlikely to represent biofilm growth, since they are removed from the biofilm nidus (i.e., bone flap). Therefore, *S. aureus* persistence in the brain and galea likely reflects continual seeding of bacteria that have detached from the bone flap as a natural process of biofilm growth and dispersal [42, 43]. This agrees with prior reports that planktonic *S. aureus* (i.e., individual bacterial cells) trigger TLR2- and caspase-1-dependent pathways [11, 25, 44]. However, it is clear that neither molecule is capable of preventing biofilm establishment or persistence, since this occurs in WT animals where both TLR2 and caspase-1 are present. The mechanisms whereby *S. aureus* is able to persist on the bone flap and chronically seed the brain and galea out to 9 months post-infection remain unknown [21]. *S. aureus* expresses over 100 virulence factors aimed at thwarting immune-mediated recognition and killing [45], suggesting that the organism utilizes a combination of virulence determinants to establish and maintain biofilm growth during craniotomy infection. Prior studies have identified several molecules that are produced by planktonic *S. aureus* that interfere with TLR2-dependent recognition, including lipase (Geh) [46], staphylococcal superantigen-like protein 3 (SSL3) [47], and molecular mimicry via blocking the Toll-interacting receptor (Tir) domain of TLR2 [48, 49]. In addition, the paired-immunoglobulin-like receptor (PIR)-B contains an inhibitory immunoreceptor tyrosine-based inhibition (ITIM) motif that upon binding *S. aureus* lipoteichoic acid, dampens pro-inflammatory cytokine production [50, 51]. In terms of caspase-1, *S. aureus* O-acetyl-transferase A (OatA) modifies peptidoglycan (PGN) following phagocytosis, rendering it more resistant to lysosomal degradation, effectively blunting caspase-1 activation and IL-1β production [52]. No information is currently available regarding the involvement of these mechanisms in facilitating *S. aureus* biofilm persistence.

One remaining question pertains to the NLR sensor that is responsible for caspase-1 activation during *S. aureus* craniotomy infection. NLRP3 was a prime candidate based on its ability to trigger caspase-1 inflammasome activation in response to *S. aureus* α-toxin [44]; however, bacterial burden and immune infiltrates in the brain and galea were similar between NLRP3 KO and WT mice. This agrees with prior work from our group using a mouse model of *S. aureus* brain abscess, where infection was localized to a defined area of the brain parenchyma, which also showed no involvement for NLRP3 [53]. In addition, *S. aureus* craniotomy infection was also ASC-independent, which differs from brain abscesses that required ASC via activation of the upstream NLR sensor absent in melanoma 2 (AIM2) [53]. Therefore, these results suggest that caspase-1 activation during *S. aureus* craniotomy infection occurs by pairing with a NLR that directly interacts with caspase-1. Only two NLRs have been identified to date that can directly bind and activate caspase-1, namely NLRP1 and NLRC4 [54]; however, neither appear to be strong candidates based on their known ligands. For example, NLRP1 recognizes *Bacillus anthracis* (*B. anthracis*) lethal toxin and NLRC4 binds to bacterial flagellin [55]. *S. aureus* is a gram-positive organism that lacks flagella and NLRC4 signals via caspase-11 to activate caspase-1, which also suggests that it is not involved in *S. aureus* recognition because our caspase-1 KO mice had functional caspase-11. Although some genes in *B. anthracis* share homology with *S. aureus*, no NLRP1 ligand has been identified to date in *S. aureus*. One study has reported that muramyl dipeptide (MDP), the minimal essential structure of bacterial PGN, is also recognized by NLRP1 [56], and cytosolic recognition of *S. aureus* is feasible since the organism has been shown to survive intracellularly [57]. Currently, the upstream sensor(s) responsible for caspase-1 activation in the context of *S. aureus* craniotomy infection remain unknown.

One interesting finding that emerged from this study was that not all biofilm infections are sensed similarly by the immune system. For example, earlier work by us and others demonstrated that *S. aureus* biofilm infection in the periphery is not affected by TLR2 loss [22, 58], whereas we show here that TLR2 is important for bacterial containment during *S. aureus* craniotomy infection. In contrast, MyD88 is critical for preventing bacterial outgrowth during both craniotomy and peripheral biofilm infections, the latter of which is partially IL-1R-dependent [9, 58, 59]. We also established a role for IL-1β during craniotomy infection by the ability of exogenous IL-1β delivery to prevent heightened bacterial burden in caspase-1 KO mice. We had expected that TLR9 would contribute to host recognition of *S. aureus* in the craniotomy infection model based on the presence of bacterial extracellular DNA (eDNA) in the biofilm matrix [60–62]. Although there was a trend towards elevated bacterial burden in the brain of TLR9 KO mice, this did not reach statistical significance. The reason why TLR9 did not play a critical role in controlling *S. aureus* biofilm infection is unclear; however, several possibilities can be considered. First, prior work from our laboratory in a mouse model of *S. aureus* catheter-associated biofilm infection also demonstrated a TLR9-independent phenotype [22], confirming the findings obtained during craniotomy infection. Second, as previously mentioned, numerous *S. aureus* virulence determinants have recently been identified that interfere with TLR2 recognition or signaling [46–49]; however, to date, no *S. aureus* inhibitors of TLR9-dependent pathways have been reported. By extension, this suggests that TLR2-mediated recognition of *S. aureus* is more relevant for bacterial clearance, since this pathway has been extensively targeted by the organism. In addition, the endosomal location of TLR9 requires phagocytic uptake to occur in order to trigger the receptor by bacterial DNA. We and others have demonstrated that staphylococcal biofilms attenuate macrophage phagocytosis [22, 61, 63], which could be another explanation for why TLR9 does not significantly impact biofilm burden, since the ligand would have limited access to TLR9 intracellularly. Nevertheless, our findings suggest key distinctions between biofilms associated with craniotomy versus other peripheral sites (i.e., orthopedic implant), which our laboratory is continuing to investigate. This is important, particularly when considering potential therapeutic options that could be applicable to a wide range of biofilm-associated infections.

## Conclusions

Collectively, these findings demonstrate that the host mounts an initial immune response to *S. aureus* craniotomy infection that is TLR2- and caspase-1-dependent, with IL-1β being a key player. However, the induction of pro-inflammatory mediators only functions to keep bacterial growth in check, as evident by heightened bacterial burdens in TLR2 and caspase-1 KO mice, but does not lead to infection clearance, since biofilms remain chronic in the WT setting where both molecules are present. This recapitulates infection in humans, where effective treatment requires biofilm removal, either by extensive debridement of the bone flap or its replacement, since endogenous immune responses are not effective. Further studies using the mouse craniotomy infection model coupled with specimens from patients with craniotomy infections should help to elucidate critical pathways of biofilm immune evasion and potential therapeutic targets in combination with antibiotic therapy.

## Supplementary information

**Additional file 1: **Characterization of IL-1β containing microparticles. (A) Scanning electron micrograph and (B) release kinetics of IL-1β loaded poly(lactide-co-glycolide) (PLGA) microparticles over a 28 day period *in vitro*.

**Additional file 2: **TLR9 does not impact *S. aureus* craniotomy infection. WT and TLR9 KO mice were sacrificed at days 3 or 7 following *S. aureus* craniotomy infection, whereupon bacterial burden in the galea, brain, and bone flap was quantified. Results were combined from 3 independent experiments (n=14 mice/group).

**Additional file 3: **NLRP3 and its adaptor ASC do not dramatically affect *S. aureus* craniotomy infection. WT, NLRP3 KO, and ASC KO mice (n=5 mice/group) were sacrificed at days 3, 7, or 14 following *S. aureus* craniotomy infection, whereupon bacterial burden in the galea, brain, and bone flap was quantified. Results were analyzed by One-way ANOVA with Tukey’s multiple comparison test (*, *p* < 0.05; **, *p* < 0.01).

## Data Availability

All data generated or analyzed during this study are included in this manuscript.

## Abbreviations

AIM2: Absent in melanoma 2
ASC: Apoptosis-associated speck-like protein containing a carboxy-terminal CARD
BSA: Bovine serum albumin
CARD: Caspase activation and recruitment domain
Casp: Caspase
CFU: Colony-forming unit
CCL2: Macrophage chemoattractant protein-1 (MCP-1)
CCL3: Macrophage inflammatory protein-1 alpha (MIP-1α)
CCL4: Macrophage inflammatory protein-1 beta (MIP-1β)
CCL5: Regulated upon activated T cell expressed and secreted (RANTES)
CCL11: Eotaxin
CNS: Central nervous system
CpG: Cytosine-phosphate-guanine
CXCL1: Keratinocyte chemokine (KC)
CXLC2: Macrophage inflammatory protein-2 (MIP-2)
CXCL9: Monokine induced by IFN-γ (MIG)
CXCL10: Interferon-inducible protein 10 kDa (IP-10)
DMEM: Dulbecco’s modified Eagle medium
EDTA: Ethylenediaminetetraacetic acid
ELISA: Enzyme-linked immunosorbent assay
FBS: Fetal bovine serum
G-CSF: Granulocyte colony-stimulating factor
Geh: *S. aureus* lipase
GM-CSF: Granulocyte-macrophage colony-stimulating factor
H&E: Hematoxylin and eosin
IL-1β: Interleukin-1β
IFN-γ: Interferon-gamma
KO: Knockout
ITIM: Immunoreceptor tyrosine-based inhibition
LIF: Leukemia inhibitory factor
LIX: CXCL5/granulocyte chemotactic protein-2
MAPK: Mitogen-activated protein kinase
M-CSF: Macrophage colony-stimulating factor
MDP: Muramyl dipeptide
MOI: Multiplicity of infection
MyD88: Myeloid differentiation factor 88
MDSC: Myeloid-derived suppressor cell
MRI: Magnetic resonance imaging
NF-κB: Nuclear factor-kB
NLR: Nucleotide oligomerization domain-like receptor
OatA: O-acetyl-transferase A
OPI: Oxalacetic acid, pyruvate, insulin
Pam3CSK4: Pam3CysSerLys4
PAMP: Pathogen-associated molecular pattern
PBS: Phosphate-buffered saline
PFA: Paraformaldehyde
PGLA: Poly(lactide-co-glycolide)
PGN: Peptidoglycan
PIR: Paired-immunoglobulin-like receptor
PVA: Polyvinyl alcohol
RBC: Red blood cell
*S. aureus*: *Staphylococcus aureus*
SEM: Scanning electron microscopy
SSL3: Staphylococcal superantigen-like protein 3
Tir: Toll-interacting receptor
TLR2: Toll-like receptor 2
TLR9: Toll-like receptor 9
TNF-α: Tumor necrosis factor-alpha
TSA: Tryptic soy agar
VEGF: Vascular endothelial growth factor
WT: Wild type
