# Supplementary material for: TLR2 and caspase-1 signaling are critical for bacterial containment but not clearance during craniotomy-associated biofilm infection
Source: J Neuroinflammation. 2020 Apr 14;17:114. doi: 10.1186/s12974-020-01793-6 (PMC7158029; doi:10.1186/s12974-020-01793-6)
Supplement: Supplementary file 1 — Additional file 1: Characterization of IL-1β containing microparticles. (A) Scanning electron micrograph and (B) release kinetics of IL-1β loaded poly(lactide-co-glycolide) (PLGA) microparticles over a 28 day period in vitro. [file 12974_2020_1793_MOESM1_ESM.pdf]

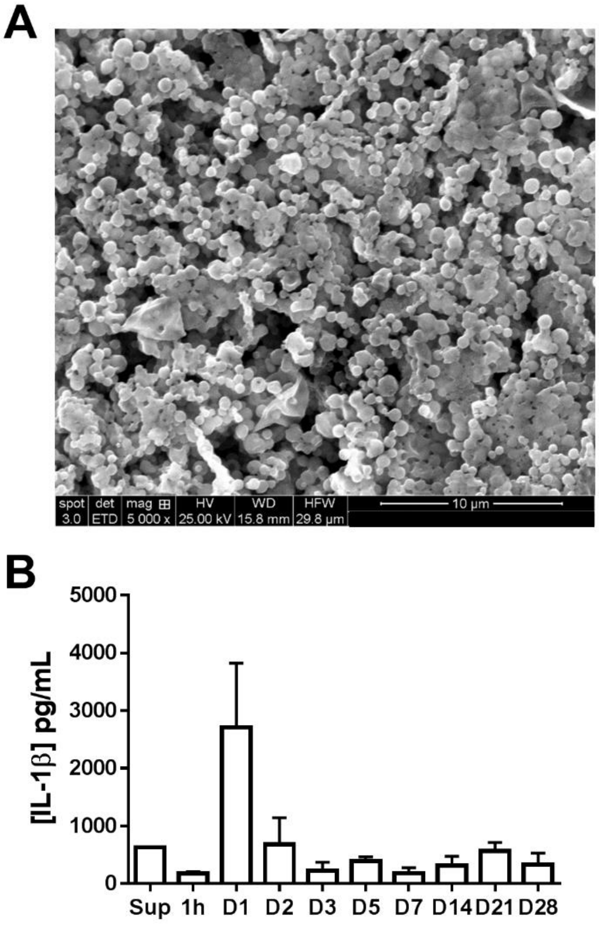

Additional File 1. Characterization of IL-1β containing microparticles. (**A**) Scanning electron micrograph and (**B**) release kinetics of IL-1β loaded poly(lactide-co-glycolide) (PLGA) microparticles over a 28 day period *in vitro* vs. non-encapsulated cytokine in the supernatant (Sup) following microparticle synthesis.
