# Supplementary material for: TLR2 and caspase-1 signaling are critical for bacterial containment but not clearance during craniotomy-associated biofilm infection
Source: J Neuroinflammation. 2020 Apr 14;17:114. doi: 10.1186/s12974-020-01793-6 (PMC7158029; doi:10.1186/s12974-020-01793-6)
Supplement: Supplementary file 2 — Additional file 2: TLR9 does not impact S. aureus craniotomy infection. WT and TLR9 KO mice were sacrificed at days 3 or 7 following S. aureus craniotomy infection, whereupon bacterial burden in the galea, brain, and bone flap was quantified. Results were combined from 3 independent experiments (n=14 mice/group). [file 12974_2020_1793_MOESM2_ESM.pdf]

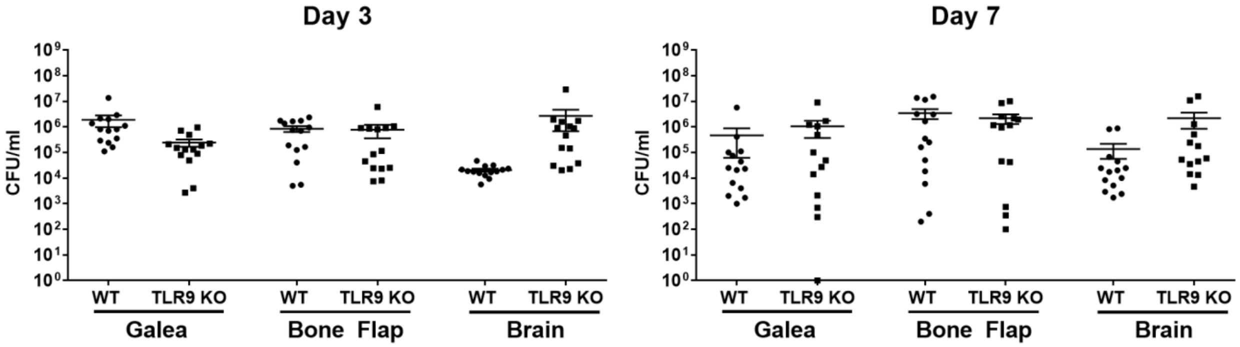

Additional File 2. TLR9 does not impact *S. aureus* craniotomy infection. WT and TLR9 KO mice were sacrificed at days 3 or 7 following *S. aureus* craniotomy infection, whereupon bacterial burden in the galea, brain, and bone flap was quantified. Results were combined from 3 independent experiments (n=14 mice/group).
