# Supplementary material for: TLR2 and caspase-1 signaling are critical for bacterial containment but not clearance during craniotomy-associated biofilm infection
Source: J Neuroinflammation. 2020 Apr 14;17:114. doi: 10.1186/s12974-020-01793-6 (PMC7158029; doi:10.1186/s12974-020-01793-6)
Supplement: Supplementary file 3 — Additional file 3: NLRP3 and its adaptor ASC do not dramatically affect S. aureus craniotomy infection. WT, NLRP3 KO, and ASC KO mice (n=5 mice/group) were sacrificed at days 3, 7, or 14 following S. aureus craniotomy infection, whereupon bacterial burden in the galea, brain, and bone flap was quantified. Results were analyzed by One-way ANOVA with Tukey’s multiple comparison test (*, p < 0.05; **, p < 0.01). [file 12974_2020_1793_MOESM3_ESM.pdf]

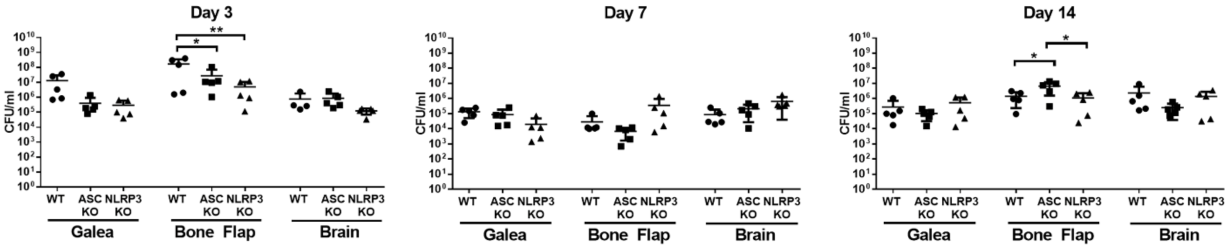

Additional File 3. NLRP3 and its adaptor ASC do not dramatically affect *S. aureus* craniotomy infection. WT, NLRP3 KO, and ASC KO mice (n=5 mice/group) were sacrificed at days 3, 7, or 14 following *S. aureus* craniotomy infection, whereupon bacterial burden in the galea, brain, and bone flap was quantified. Results were analyzed by One-way ANOVA with Tukey's multiple comparison test (*, $p < 0.05$; **, $p < 0.01$).
